# Retroviral Gene Vectors Show Clear Target Preferences

**DOI:** 10.1371/journal.pbio.0020443

**Published:** 2004-11-23

Despite some high-profile setbacks in gene therapy over the past few years, scientists have not lost hope that targeted gene transfer will one day treat a wide range of acquired and congenital diseases. After two young gene therapy patients developed a leukemia-like disorder last year—apparently because the viral vector used to carry the corrective gene activated a cancer-causing gene—researchers redoubled their efforts to develop safer, more effective retroviral gene delivery methods. Such efforts depend on understanding how and where retroviral vectors integrate into the genome.

In a new study, Cynthia Dunbar and colleagues describe a nonhuman primate model that mimics gene therapy protocols in humans, and report the integration biases of two classes of retroviral vectors being developed for clinical trials. The retroviruses, the authors show, display clear preferences that not only suggest different genomic integration mechanisms but also have different implications for safety.

Retroviral gene therapy exploits a retrovirus's skill at entering a cell, infiltrating its genome, and hijacking its molecular machinery for its own reproductive advantage. In gene therapy, therapeutic genes largely take the place of viral genes, and so the “infected” cell churns out beneficial gene products, not viruses. Before a retrovirus can integrate into a host cell, however, it must copy its genome—which is encoded in RNA—into DNA, so the cell's copying machinery will recognize it. After generating this “pre-integration complex,” the virus must access the cell's chromosomal DNA, which lies behind a nuclear barrier. Different retroviruses accomplish this task in different ways.

Lentiviruses—which include AIDS and SIV (simian immunodeficiency virus)—can infect nondividing cells simply by slipping through nuclear pores. Oncoretroviruses—such as murine leukemia virus, or MLV, the vector type used for the vast majority of previous clinical trials, including the trial complicated by leukemia in two patients—must wait until the nuclear membrane dissolves during cell division. Once integrated into the host genome, the provirus—and its therapeutic gene—will persist through each new cell division—a trait that underlies its usefulness as a vector as well as its risk. Retroviruses that insert near proto-oncogenes can activate these genes and set the cell on the path to tumorigenesis. Until recently, researchers assumed this risk was extremely low because retroviral integration was thought to be random—an assumption recently undercut by a number of studies that mapped retroviral integration in different cell lines.

Dunbar and colleagues take these studies a step further by mapping the integration patterns of MLV and SIV vectors in hematopoietic stem cells (HSCs) of rhesus monkeys. HSCs are the cells typically used to carry these vectors for therapeutic applications involving any of the cell types, such as red blood cells, produced by the bone marrow. The monkeys had received infusions of HSCs carrying either the SIV or MLV vectors between six months and six years earlier. Two types of white blood cells (granulocytes and mononuclear cells) were harvested from the monkeys and evaluated for proviral insertion sites.

Of nearly 1,000 integration sites identified, 760 could be mapped to unique corresponding sites in the human genome (432 MLV and 328 SIV). While both MLV and SIV vectors tended to integrate within genes, MLV showed a strong preference for the starting end of genes, most likely to result in gene activation. In contrast, SIV showed a preference for genomic regions of high gene density, but not for specific sites within a gene. Surprisingly, MLV targeted one gene—known previously to be involved in spontaneous leukemias and in murine retroviral oncogenesis—seven times, a “highly nonrandom” result suggesting that such insertions may occur far more often than previously thought. About 40 genes, including seven known oncogenes, were targeted more than once by one or both vectors. Such differences, Dunbar and colleagues note, “likely reflect the vectors' distinct mechanisms for accessing DNA and integrating,” which could in turn affect their risk of causing insertional mutagenesis. Even though the vectors tend to integrate nonrandomly and can target oncogenes, however, none of the monkeys showed signs of ill effects such as leukemia.

But before any widespread applications of retroviral gene therapy can proceed, Dunbar and colleagues argue, potential risks of proviral insertion must be assessed in the specific cell types associated with different gene therapies. And with a model for long-term, genome-wide retroviral integration analysis that mimics human gene therapy protocols, the authors have made an important contribution toward that end.

